# CUT BARB (an Acronym for Fishhook Injuries): Illustrated by the Extraction of a Fishhook From the Auricle Using the Advance-and-Cut Technique

**DOI:** 10.7759/cureus.30146

**Published:** 2022-10-10

**Authors:** Mohammed Abrahim

**Affiliations:** 1 Division of Emergency Medicine, Department of Family Medicine, McMaster University, Hamilton, CAN; 2 Emergency Department, Milton District Hospital, Milton, CAN

**Keywords:** removal of foreign body, auricular injury, wilderness medicine, rural emergency medicine, recreational sports, recreational activities, fishhook, fishhook injury, fishhook removal

## Abstract

Fishhook injuries are common within fishing communities. The embedded barb in the tissue prevents the hook from being pulled out until the barb is disengaged. Although the majority of injuries are minor, occasionally fishhook injuries can be serious. Herein, the author developed a novel acronym: CUT BARB (C: consult immediately for critical areas, U: underlying structure injury, T: tetanus immunization, B: barb shape and size, A: antibiotic prophylaxis, R: radiology imaging, B: bait or lure fragments). The aim of the acronym is to aid emergency department (ED) physicians in conducting a comprehensive clinical assessment and reaching a prompt therapeutic decision for embedded fishhook injuries. Additionally, the author is demonstrating the technique via the extraction of a medium-sized single-barbed fishhook embedded in the left auricle of a 60-year-old female using the advance-and-cut technique.

## Introduction

Fishing is a popular recreational activity globally, and fishhook injuries are common, particularly during the summer in fishing communities [1]. Barbless hooks are easily removed and are rarely present to the emergency department (ED). However, a barb embedded in tissue prevents the hook from being pulled out until the barb is disengaged, so its removal requires skill. Despite the majority of fishhook injuries being minor in nature, some serious injuries involving the eyes, arteries, genitals, neck, or airway do occur [2]. Additionally, fishhook injuries require assessment of the involved underlying structure prior to removal. The primary extraction techniques are: advance and cut; string-yank; simple retrograde; and needle cover. The number of fishhook barbs and the location of the embedded hook dictate the method of removal [1]. The advance-and-cut method is the most commonly used and successful method to date [2].

The author presents a case of the extraction of an embedded single-barbed fishhook from the left auricle of a 60-year-old female auricle using the advance-and-cut technique, in addition to presenting a novel acronym, CUT BARB (C: consult immediately for critical areas, U: underlying structure injury, T: tetanus immunization, B: barb shape and size, A: antibiotic prophylaxis, R: radiology imaging, B: bait or lure fragments), for fishhook injury assessment in the ED.

## Technical report

**Figure 1:**
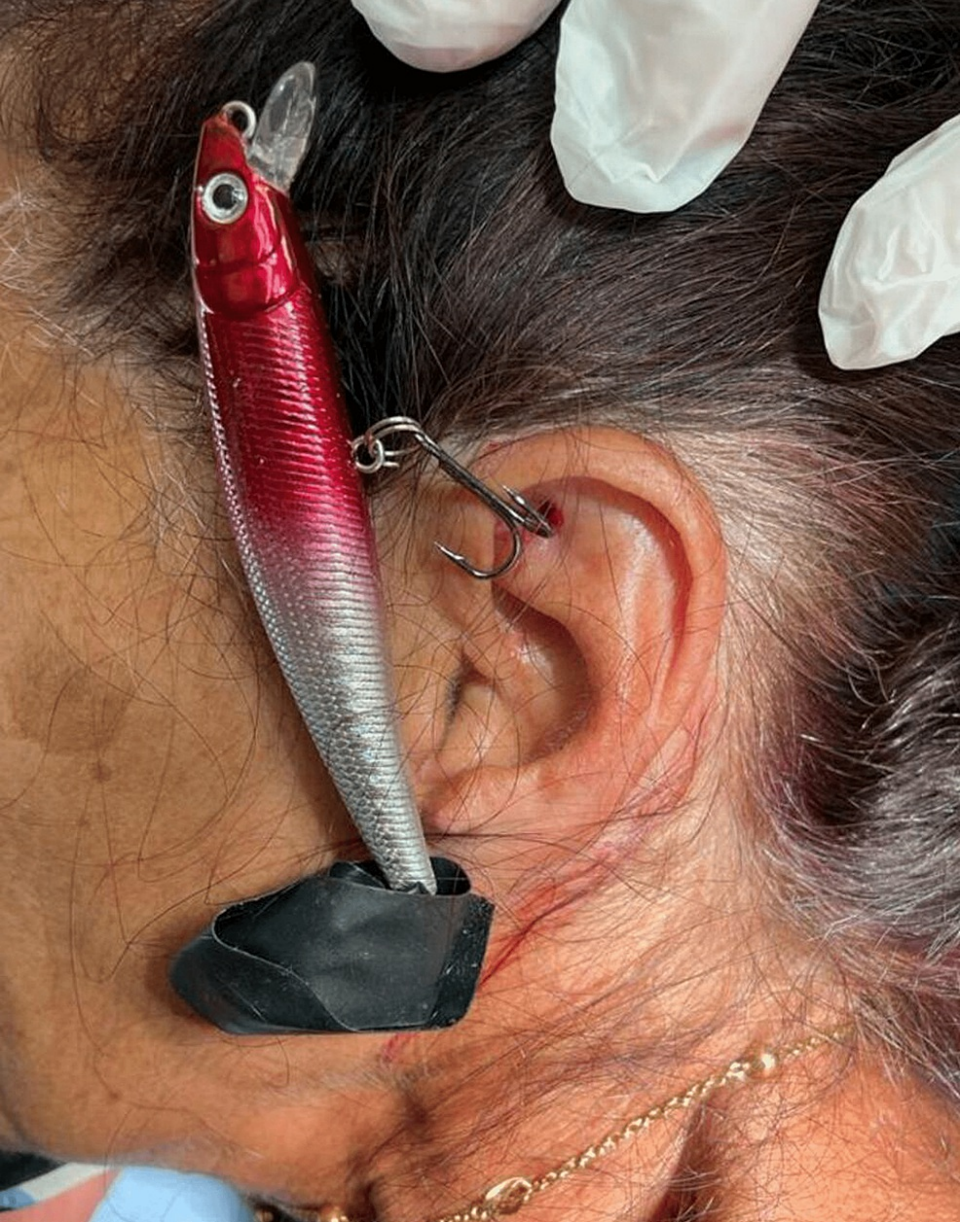
A large lure attached to a single-barbed and treble fishhook with one hook embedded in the patient’s left auricle.

A previously healthy 60-year-old woman presented to the ED shortly after a fishhook was accidentally embedded in her left auricle while fishing with her spouse. She cut the fishing line immediately but could not remove the embedded fishhook or the attached lure and presented to our ED. On examining the fishhook, it was found to be triple-hooked and single-barbed with only one hook penetrating through the upper pole of the auricle with its tip close to the skin surface. The hook was attached to a big lure with no attached bait (Figure 1).

**Figure 2:**
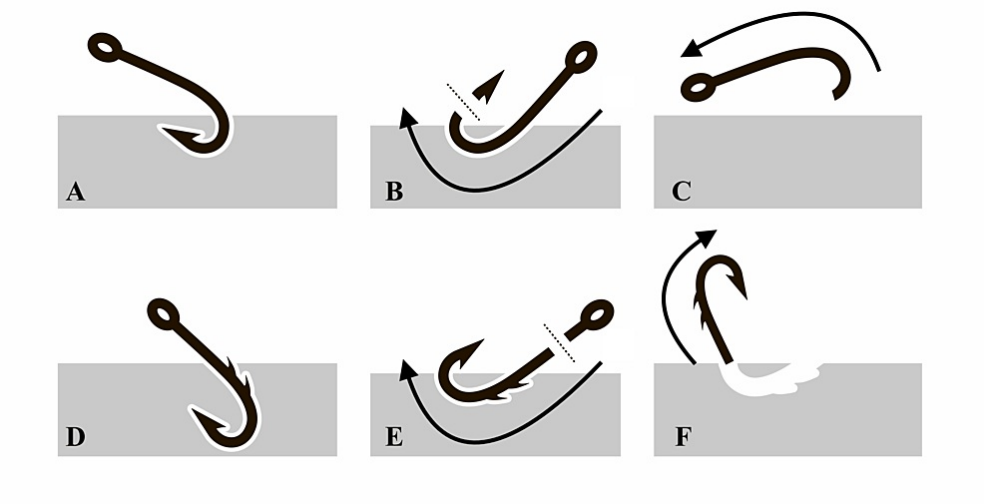
Fishhook removal using advance-and-cut technique. Single-barbed (A, B, C) and multi-barbed (D, E, F) [1].

Because the hook was single-barbed and its tip was close to the skin surface, the decision was made to use the advance-and-cut technique for single-barbed hooks (Figure 2A-2C).

The site was cleaned using a chlorhexidine solution. 0.5 ml of lidocaine 1% without epinephrine was infiltrated around the fishhook. The hook was advanced out of the exit wound, exposing the barb. Multiple tools were used to attempt to cut the barb but, because of its relatively large size, a larger tool was required. We used a pair of pliers. After cutting off the barb, we retracted the fishhook backward out of the inlet wound (Video 1).

**Video 1:** Extraction of an embedded single-barbed fishhook from the left auricle using advance-and-cut technique.

While cutting off the barb, the physician and nursing staff wore eye protection to prevent anyone else from getting injured. A wound dressing with local antibiotic ointment was applied. Because the patient’s tetanus immunization status was not up-to-date, she received a tetanus toxoid booster dose. Additionally, due to auricular cartilage involvement, prophylactic oral antibiotics were prescribed. The patient was discharged and is expected to make a full recovery.

## Discussion

**Figure 3:**
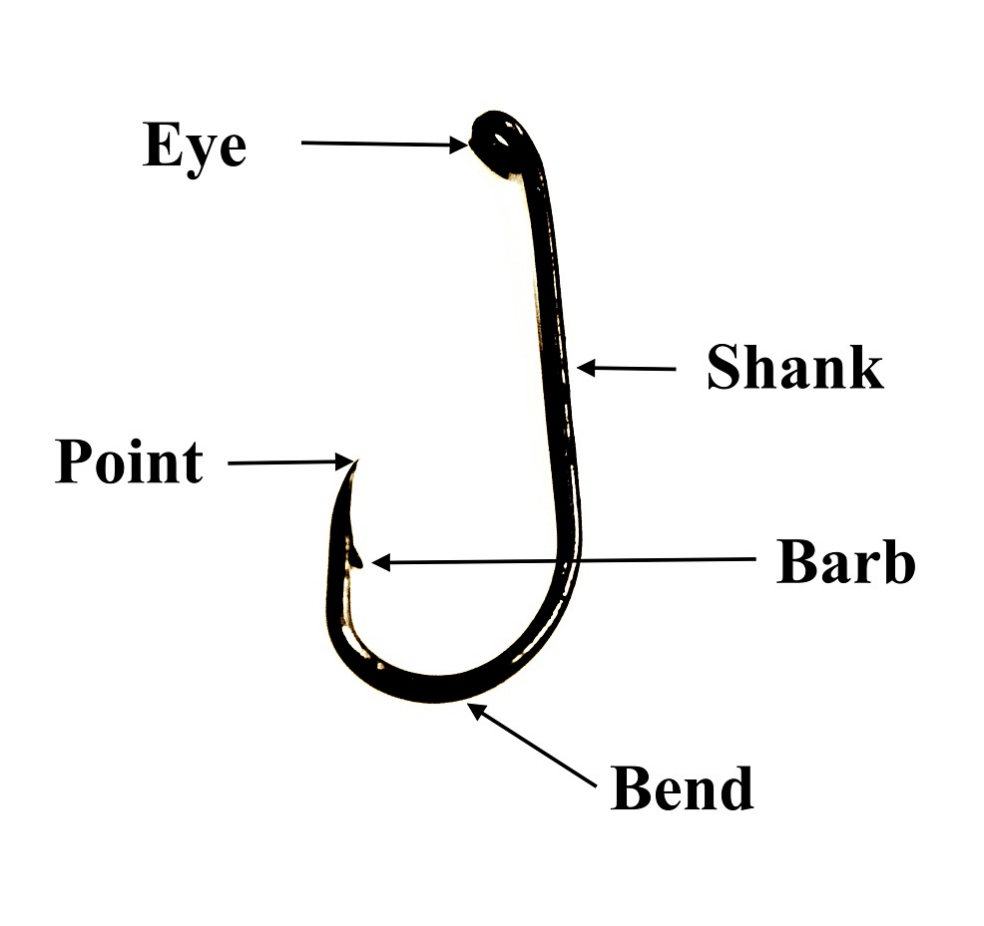
The structure and parts of the fishhook.

Extraction of embedded fishhooks in the ED can be challenging due to the presence of barb(s) and requires technical skills plus familiarity with fishhook structure (Figure 3).

There are several techniques for the extraction of embedded fishhooks. The primary four techniques are: advance and cut; string-yank; simple retrograde; and needle cover [1]. Furthermore, the advance-and-cut method is the most commonly used and successful method to date [1]. The advance-and-cut technique is selected when fishhook tips are close to the skin surface and where there are no blood vessels, nerves, tendons, or bones in the way.

The advance-and-cut technique has two common variations depending on whether the fishhook is single or multi-barbed. For a single-barbed hook (Figure 2A-2C), the tip of the hook is advanced through the skin, then the barb is cut off when fully exposed, and then the shank is pulled back out of the entry wound [1]. Unlike in multi-barbed hooks (Figure 2D-2F), the tip of the hook is advanced through the skin until the barb is exposed, then the eye of the hook is cut off and the shank is completely advanced through the exit wound [1].

Recently, Pakniyat et al. introduced a modification of the advance-and-cut technique which was described as the advanced without-cut and retrograde fishhook removal technique [2], in which the fishhook is advanced through the wound until the barb passes the skin, then the barb is clamped and bent over the body of the bent part, transforming the fishhook into a barbless hook, then the fishhook is retracted without cutting [2].

In the string-yank method, a string is tied around the shank of the hook, then downward pressure is applied to the shank while the string is pulled parallel to the shank [3]. In the simple retrograde method, the shank of the fishhook is pushed downwards and then pulled back [3]. In the needle cover method, a needle tip is inserted to cover the embedded barb, then the hook is pulled out. Finally, the fishhook could be removed by using a small incision along the shank until the scalpel reaches the barb, and then the full intact hook is pulled out through the incision [3].

A retrospective review of fishhook injuries between 2013 and 2015 conducted within a fishing community in Canada found that most injuries occurred to the hands and during the summer [4]. The most commonly used, and most successful extraction technique to date, has been the advance-and-cut. Within the review previously mentioned, imaging was undertaken in 20% of cases (plain radiographs). Furthermore, more than half of the cases received prophylactic antibiotics, with cephalexin being the most commonly prescribed [4].

To search for fishhook injuries of the ears, we conducted a literature review by searching the PubMed electronic database published in MEDLINE. The following keywords were searched: fishhook, ear. The search returned only two relevant case reports of fishhook removal from the ear [5,6]. In one case, a single-barbed, double-hooked fishhook was embedded in the upper part of the auricle and was removed surgically by a controlled incision around the barb under local anesthesia and prophylactic antibiotics were prescribed [5]. The second case highlights the importance of assessing the depth of penetration and the potential underlying structural injuries because the fishhook pierced through the left auricle and also penetrated the underlying parotid gland, causing a chronic glandular scar [6].

**Figure 4:**
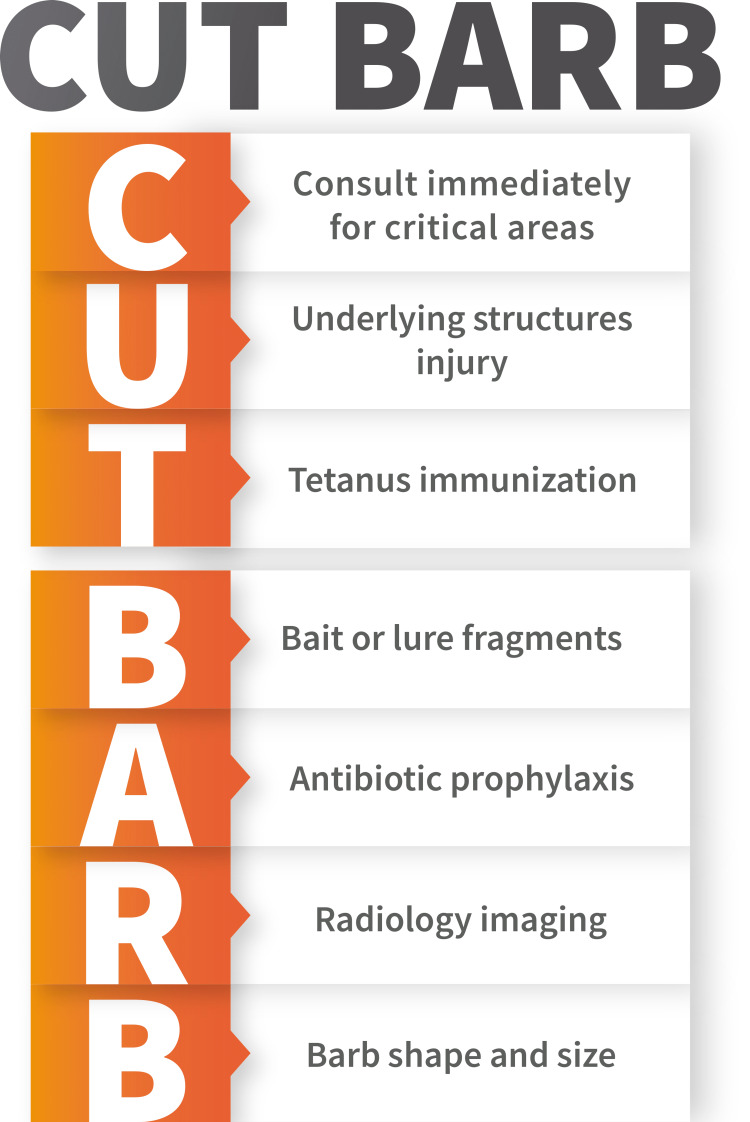
CUT BARB acronym for embedded fishhook injuries in the ED.

The author herein is introducing the novel acronym: CUT BARB (Figure 4) as a systematic approach to aid emergency physicians in performing comprehensive clinical assessments of embedded fishhook injuries.

CUT BARB 

**C**onsult immediately for fishhook injuries involving critical areas such as the eyes, eyelids, arteries (for example, carotid or radial), genitalia, neck, and airway, or when a hook is embedded very near to any of these structures. The area should be covered with a sterile dressing, and the appropriate specialty should be consulted. A literature review of ocular fishhook injuries concluded that such injuries carry a great risk of severe irreversible complications. Therefore, the authors of the aforementioned review recommended eye protection for fishermen, including children [7]. Furthermore, airway injuries could occur, especially in children [8,9]. Eley et al. reported a case of a 13-month-old boy who chewed on a fishhook at home, which got embedded in his tongue [8]. Additionally, Raveenthiran also reported a case of a six-year-old boy with an embedded fishhook injury to the soft palate [9]. It is important to emphasize that not all fishhook injuries can be removed in the ED.

**U**nderlying deep structure injury assessment is an integral element in fishhook injury assessment. Injuries to bone, cartilage, muscle, tendons, or nerves could require further imaging, specialist consultation, or antibiotic prophylaxis.

**T**etanus immunization status should always be assessed and a tetanus toxoid booster is administered if deemed necessary.

**B**arb shape and size dictate the choice of the technique used in the advance-and-cut method. In the case of single-barbed fishhooks, as in the case reported here, the physician should advance the fishhook through the skin to expose the barb and then cut off the barb. The remaining hook is backed and pulled out through the entry wound (Figure 2A-2C). For multiple-barbed fishhooks, advance the fishhook through the skin exit wound, then cut off the eye of the fishhook. Then pull the remaining portion of the fishhook through the exit wound (Figure 2D-2F).

**A**ntibiotic prophylaxis is generally not indicated. However, to date, there are no clinical trials investigating antibiotic therapy for fishhook injuries [1]. Uncomplicated minor fishhook injuries could be managed with only local antibiotics until healing is established. At this stage, patients should be discharged from the ED and advised to return if any signs of infection appear. Patients should receive prophylactic antibiotics for deeply embedded fishhooks involving deep structures or those that are contaminated. This is a particularly important consideration in immune-compromised patients.

**R**adiology imaging is indicated only in selected cases when: bone involvement is suspected; radiopaque foreign body (FB); or to identify the hook type in a completely embedded hook.

**B**ait or lure fragments could be driven into the wound with the fishhook. Physicians should not only remove the embedded barb but also consider associated bait or barb FBs.

## Conclusions

Embedded fishhook injuries continue to present themselves to emergency departments worldwide. The present paper introduces the novel acronym, CUT BARB, as a systematic approach to aid emergency physicians in performing comprehensive clinical assessments of embedded fishhook injuries. Additionally, the manuscript presents a case of the extraction of an embedded single-barbed fishhook from the auricle using the advance-and-cut technique.
